# Candidate Human Genetic Polymorphisms and Severe Malaria in a Tanzanian Population

**DOI:** 10.1371/journal.pone.0047463

**Published:** 2012-10-29

**Authors:** Alphaxard Manjurano, Taane G. Clark, Behzad Nadjm, George Mtove, Hannah Wangai, Nuno Sepulveda, Susana G. Campino, Caroline Maxwell, Raimos Olomi, Kirk R. Rockett, Anna Jeffreys, MalariaGen Consortium, Eleanor M. Riley, Hugh Reyburn, Christopher Drakeley

**Affiliations:** 1 Joint Malaria Programme, Kilimanjaro Christian Medical Centre, Moshi, Tanzania; 2 Faculty of Infectious and Tropical Diseases, London School of Hygiene and Tropical Medicine, London, United Kingdom; 3 Faculty of Epidemiology and Population Health, London School of Hygiene and Tropical Medicine, London, United Kingdom; 4 Wellcome Trust Sanger Institute, Hinxton, Cambridge, United Kingdom; 5 Wellcome Trust Centre for Human Genetics, University of Oxford, Oxford, United Kingdom; Sudbury Regional Hospital, Canada

## Abstract

Human genetic background strongly influences susceptibility to malaria infection and progression to severe disease and death. Classical genetic studies identified haemoglobinopathies and erythrocyte-associated polymorphisms, as protective against severe disease. High throughput genotyping by mass spectrometry allows multiple single nucleotide polymorphisms (SNPs) to be examined simultaneously. We compared the prevalence of 65 human SNP's, previously associated with altered risk of malaria, between Tanzanian children with and without severe malaria. Five hundred children, aged 1–10 years, with severe malaria were recruited from those admitted to hospital in Muheza, Tanzania and compared with matched controls. Genotyping was performed by Sequenom MassArray, and conventional PCR was used to detect deletions in the alpha-thalassaemia gene. SNPs in two X-linked genes were associated with altered risk of severe malaria in females but not in males: heterozygosity for one or other of two SNPs in the G6PD gene was associated with protection from all forms of severe disease whilst two SNPs in the gene encoding CD40L were associated with respiratory distress. A SNP in the adenyl cyclase 9 (ADCY9) gene was associated with protection from acidosis whilst a polymorphism in the IL-1α gene (IL1A) was associated with an increased risk of acidosis. SNPs in the genes encoding IL-13 and reticulon-3 (RTN3) were associated with increased risk of cerebral malaria. This study confirms previously known genetic associations with protection from severe malaria (HbS, G6PD). It identifies two X-linked genes associated with altered risk of severe malaria in females, identifies mutations in ADCY9, IL1A and CD40L as being associated with altered risk of severe respiratory distress and acidosis, both of which are characterised by high serum lactate levels, and also identifies novel genetic associations with severe malaria (TRIM5) and cerebral malaria(IL-13 and RTN3). Further studies are required to test the generality of these associations and to understand their functional consequences.

## Introduction

In spite of recent evidence of successful control in some countries [1], [2], [3], malaria still constitutes a major cause of child morbidity and mortality, especially in sub Saharan Africa [4], [5]. Although the number of deaths from malaria remains high, the absolute risk of any particular infection progressing to severe disease is less than 1%, even in young children who appear to have similar, often low, levels of acquired immunity [6]. The risk factors for severe disease, and the reasons for the wide variation in clinical manifestations of malaria among children who appear to exhibit similar risks for severe disease, are poorly understood. This unexplained variation in disease severity and syndromic phenotype constitutes a major challenge to our understanding of the disease, its treatment and control.

Although parasite diversity, host age, acquired immunity and overall health status may all influence the risk of severe disease complications, it has been estimated that 25% of this risk can be accounted for by variations in host genotype [7]. The different geographic distributions of sickle-cell disease, α-thalassaemia, glucose-6-phosphate dehydrogenase (G6PD) deficiency, ovalocytosis, and the Duffy-negative blood group are examples of the general principle that different populations have evolved different genetic variants to protect against malaria (see [8] for a review). The most striking example is the beta-globin HBB gene, in which three different coding SNPs confer protection against malaria: Glu6Val (HbS), Glu6Lys (HbC), and Glu26Lys (HbE). The HbS allele is common in Africa but rare in Southeast Asia, whereas the opposite is true for the HbE allele. In Tanzania, strong associations have been described between malaria transmission intensity and polymorphisms of both the HbS and alpha-thalassaemia genes [9]. In addition to the sickle polymorphism (HbS) [10], G6PD (reviewed in [11]), and ABO blood group [12], [13], a number of other traits have been proposed for the reduced risk of severe malaria. Consistent with the view that severe malaria disease is, at least in part, an inflammatory process mediated by disordered immune responses [14] many of these traits are polymorphisms in genes that are relevant to immunity and inflammation such as the tumor necrosis factor (TNF, MHC class III region, reviewed in [15], Toll-like receptors (TLR-4,9) [16], CD40 ligand (CD40L) [17], the interferon gamma (IFNG) (reviewed in [18]), and the Nitric oxide synthase type 2 (*NOS2*A) genes (reviewed in [19]).

Here we test a panel of 65 SNPs, previously linked to altered risk of malaria or other infectious diseases, including the HbS and ABO polymorphisms, for association with severe malaria, and with the various clinical presentations of severe malarial disease. Our study is the first to comprehensively survey candidate SNPs in Tanzanians resident in a hyperendemic area of malaria with a high incidence of severe malaria disease and reveals X-linked polymorphisms that affect risk of severe disease in females but not in males, as well as polymorphisms in genes associated with inflammation, antigen processing and T cell function.

## Methods

### Ethics Statement

All DNA samples were collected and genotyped following signed and informed written consent from a parent or guardian. Ethics approval for all procedures was obtained from both LSHTM (# 2087) and the Tanzanian National Institute of Medical Research (NIMR/HQ/R.8a/Vol. IX/392).

### Participants, Materials and Methods

#### Study participants

The study was conducted in Teule district hospital, Muheza, and surrounding villages in Muheza district, Tanga region, Tanzania, where mortality in children under 5 is estimated at 165 per 1000 (Tanzanian census 2002). Transmission of *P. falciparum* is intense (50–700 infected bites/person/year) and perennial, with two seasonal peaks. The community prevalence of *P. falciparum* in children aged 2–5 years in the study area was recorded as 88.2% in 2002 [20].

Cases were recruited during a one-year period between June 2006 and May 2007 as part of a larger study of severe febrile illness in children [detailed in [21], [22]]. Among the children in the parent study, 509 cases of severe malaria were randomly sampled from children aged 6 months to 10 years meeting any one of the following eligibility criteria; history of 2 or more convulsions in the previous 24 hrs, prostration (unable to sit unsupported if <9 months of age or unable to drink at any age), reduced consciousness (Blantyre Coma scale<4), respiratory distress (deep breathing or indrawing of the lower chest wall), visible jaundice, severe anaemia (Hb <5 g/dL), acidosis (blood lactate ≥5 mmol/L) or hypoglycaemia (blood glucose <2.5 mmol/L). Parasite infection was confirmed by microscopic examination of Giemsa-stained thick blood films by two independent microscopists. Participants with known chronic medical conditions were excluded. Area of residence and ethnic origins of both parents were recorded from information provided by the caretaker of each child.

Controls were recruited during a 4 week period in August 2007 and were matched with cases for area of residence (ward), ethnicity and age, using household census data. Study participants resided in 33 wards surrounding Muheza town. The participants had a median age of ∼2.6 years and belonged, predominantly, to one of eight ethnic groups (Table 1).

**Table 1:** Baseline and clinical characteristics of cases and controls.

	Controls (n = 480)		Cases (n = 507)	
*Age* * *(median, range)*	(2.9)	(0.9–10.9)	(1.7)	(0.2–10.0)
*Gender - Female*	258	53.8%	236	46.5%
*Ward*				
Mtindiro	40	8.3%	49	9.7%
Kwafungo	32	6.7%	43	8.5%
Mkata	32	6.7%	32	6.3%
Kwedizinga	30	6.3%	31	6.1%
Songa	25	5.2%	25	4.9%
Nkumba	21	4.4%	27	5.3%
Segera	23	4.8%	23	4.5%
Maramba	22	4.6%	21	4.1%
Mhinduro	20	4.2%	20	3.9%
Potwe	20	4.2%	20	3.9%
Kilulu	19	4.0%	19	3.7%
Lusanga	18	3.8%	18	3.6%
Kicheba	15	3.1%	18	3.6%
Ngomeni	16	3.3%	16	3.2%
*Ethnicity*				
Mzigua	159	33.1%	149	29.4%
Wasambaa	132	27.5%	131	25.8%
Wabondei	78	16.3%	79	15.6%
Chagga	50	10.4%	47	9.3%
Mmbena	23	4.8%	25	4.9%
Other	5	1.0%	39	7.7%
Mngoni	18	3.8%	19	3.7%
Pare	15	3.1%	18	3.6%
*Clinical phenotype*				
Any severe malaria	-	-	507	100%
Any SMA**	-	-	247	48.7%
Any CM	-	-	99	19.5%
Both SMA+CM	-	-	41	8.1%
Any RD	-	-	146	28.8%
Acidosis***	-	-	291	57.4%

* in months, SMA = severe malarial anaemia, CM = cerebral malaria, RD = respiratory distress,

** hb<5 gdl,

*** Blood lactate>5 mmol/l.

#### Sample collection & preparation

Approximately 3 ml of venous blood was collected from each participant into an EDTA vacutainer. A blood film was prepared and haemoglobin levels were measured by Hemocue (Hemocue™, Anglholm, Sweden). For children in the control group, those with haemoglobin levels of <11 g/dL and those with a positive blood film were excluded from genetic analysis and were referred to the nearest health facility where they were treated according to Tanzanian Ministry of Health guidelines. Samples were centrifuged at 5000 rpm for 5 minutes and the plasma removed and stored for future analysis. DNA was extracted and purified from the blood cell pellet using a Nucleon kit (http://www.tepnel.com) according to the manufacturer's instructions.

#### Sample genotyping

Genomic DNA samples underwent whole genome amplification through either Primer Extension Pre-amplification (PEP) [23]or Multiple Displacement Amplification (MDA) [24], before genotyping on a Sequenom MassArray genotyping platform [25], [26]. Sixty-five candidate SNPs (see supplementary Table 1) were selected for typing based on an extensive review of the published data and on emerging data from the MalariaGEN consortium (http://www.malariagen.net). The selected SNPs included Haemoglobin variant S (HbS) (rs334) as an anticipated positive control. The α^3.7^-thalassaemia deletion was typed separately by PCR [27].

#### Phenotypic definition

For analysis, subjects were defined as having had cerebral malaria (CM) if their Blantyre coma score was less than 3 on presentation to hospital. A second phenotypic subset of severe malarial anaemia (SMA) was defined as those subjects having had a haemoglobin concentration of less than 5 g/dL. Patients with both CM and SMA were included in the analysis for both phenotypes. Participants with co-existing severe illnesses diagnosed on admission, or with known chronic medical conditions unrelated to a severe malarial infection, were excluded. Other phenotypes of interest in severe malaria cases included respiratory distress and acidosis (blood lactate greater than > = 5 mmol/L). Because of sample size limitations, we do not present an analysis by ethnic groups or ward of residence.

#### Statistical analysis

Genotypic deviations from Hardy-Weinberg equilibrium (HWE) were assessed by chi-square tests. SNPs were excluded from analysis if they had at least 10% of genotype calls missing or there was significant deviation from HWE (p<0.001) in controls. Case-control association analysis using SNP alleles/genotypes was undertaken by logistic regression and included the covariates: age, gender, ethnic group (or ward). We modelled the SNP of interest assuming several related genotypic mechanisms (additive, dominant, recessive, heterozygous advantage and general models) and reported the minimum p-value from these correlated tests. All haplotypes were phased using an expectation-maximisation algorithm [28]. All analyses were performed using the R statistical package (http://www.r-project.org). Performing multiple statistical tests leads to inflation in the occurrence of false positives and using a permutation approach that accounted for correlation between markers and tests, we estimated a p-value of ≤0.02 to be statistically significant.

## Results

Seven SNPs were removed from the analysis because they were either monomorphic (rs2814778, rs33950507, rs1799969, rs5743611, rs4986791, X_80140046), deviated from HWE in controls (rs1800750) or had high rates (>10%) of missing genotype calls (rs1800750), leaving 58 SNPs which could be analysed for their association with severe malaria.

**Figure 1:**
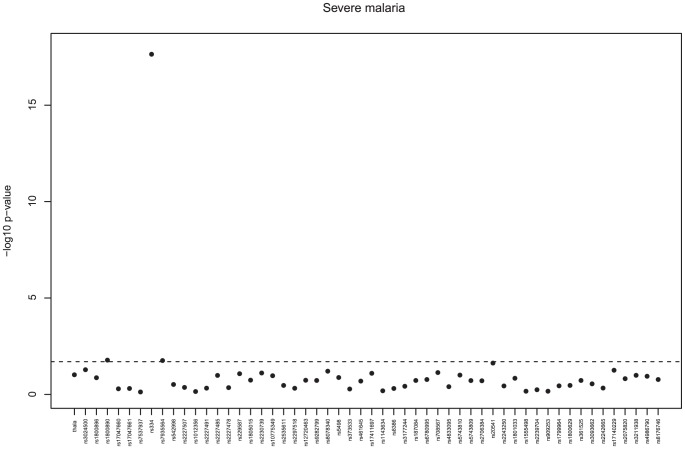
Severe malaria, Minimum p-values from tests of association for the autosomal SNPs. genotypic tests of dominant, recessive, general, heterozygous advantage, and additive models, adjusted for HbS and ethnicity; in this analysis controls include uncomplicated malaria cases; the dashed line represents a p-value of 0.002.

**Figure 2:**
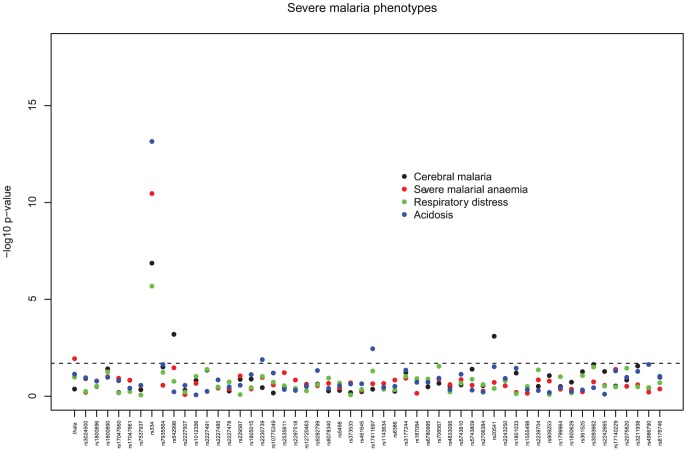
Severe malaria phenotypes; minimum p-values from tests of association for the autosomal SNPs. genotypic tests of dominant, recessive, general, heterozygous advantage, and additive models, adjusted for ethnicity; the dashed line represents a p-value of 0.002.

Figure 1 shows the minimum p-values from the genotypic tests applied to the autosomal SNPs, and confirms that the sickle cell (HbS) polymorphism (rs334) was significantly associated with protection from severe malaria (odds ratio (OR) AS vs AA/SS 0.055, 95% CI 0.022–0.140, p<3e-18). Carriage of the HbS trait was also associated with protection from each of the different clinical phenotypes of severe malaria (Table 2 & Figure 2). Individuals who had α^3.7^-thalassaemia deletion (in the additive model) were 30% less likely than individuals without this polymorphism to develop severe malarial anaemia (p = 0.01) but were not significantly protected against other forms of severe disease. There was no statistical interaction between carriage of the HbS trait and the α^3.7^ deletion and risk of any severe malaria complications (Table 3, p>0.4).

**Table 2:** Results of association between selected SNP and syndromes of severe malaria.

Phenotype	SNP	Gene	Maj/Min	Con MAF	CaseMAF	Comparison	O	LCL	UCL	P
SM	rs1800890	IL10	A/T	0.240	0.207	Additive T	0.747	0.588	0.949	0.0165
SM	rs334	HBB	A/T	0.083	0.016	AT vs. AA/TT	0.055	0.022	0.140	2.28E-18
SM	rs7935564	TRIM5	G/A	0.412	0.456	Additive A	1.273	1.042	1.555	0.0176
CM	rs334	HBB	A/T	0.083	0.000	AT vs. AA/TT	0.000	NA	NA	1.35E-07
CM	rs542998	RTN3	T/C	0.363	0.495	Additive C	1.781	1.276	2.487	0.0006
CM	rs20541	IL13	C/T	0.194	0.324	Additive T	2.020	1.346	3.030	0.0008
SMA	Thala	thala	A/B	0.319	0.251	Additive B	0.703	0.533	0.927	0.0116
SMA	rs334	HBB	A/T	0.083	0.021	AT vs. AA/TT	0.047	0.011	0.196	3.47E-11
RD	rs334	HBB	A/T	0.083	0.007	AT vs. AA/TT	0.085	0.020	0.361	2.08E-06
Acid	rs334	HBB	A/T	0.083	0.012	AT vs. AA/TT	0.021	0.003	0.150	7.07E-14
Acid	rs2230739	ADCY9	A/G	0.077	0.057	AG vs. AA/GG	0.516	0.302	0.882	0.0129
Acid	rs17411697	IL1A	G/T	0.156	0.222	GT/TT vs. GG	1.681	1.185	2.383	0.0035
										
SM – M	rs1050829	G6PD	T/C	0.371	0.366	C vs. T	1.003	0.667	1.509	0.9885
SM – F	rs1050829	G6PD	T/C	0.400	0.340	CT vs. CC/TT	0.437	0.289	0.660	6.71E-05
SM – M	rs1050828	G6PD	C/T	0.193	0.146	T vs. C	0.770	0.458	1.293	0.3228
SM – F	rs1050828	G6PD	C/T	0.211	0.174	CT vs. CC/TT	0.514	0.323	0.819	0.0046
SMA – M	rs1050829	G6PD	T/C	0.371	0.389	C vs. T	1.055	0.643	1.729	0.8325
SMA – F	rs1050829	G6PD	T/C	0.400	0.295	CT vs. CC/TT	0.278	0.154	0.501	8.64E-06
SMA – M	rs1050828	G6PD	C/T	0.193	0.170	T vs. C	0.926	0.500	1.713	0.8052
SMA – F	rs1050828	G6PD	C/T	0.211	0.139	CT vs. CC/TT	0.322	0.159	0.650	0.0008
RD – M	rs3092945	CD40LG	T/C	0.223	0.189	C vs. T	0.793	0.377	1.667	0.5372
RD – F	rs3092945	CD40LG	T/C	0.205	0.313	CC vs. CT/TT	5.270	1.491	18.621	0.0096
RD – M	rs1126535	CD40LG	T/C	0.209	0.237	C vs. T	1.301	0.634	2.670	0.4755
RD – F	rs1126535	CD40LG	T/C	0.238	0.159	CT vs. CC/TT	0.331	0.143	0.766	0.0062
RD – M	rs1050829	G6PD	T/C	0.371	0.458	C vs. T	1.369	0.748	2.505	0.3101
RD – F	rs1050829	G6PD	T/C	0.400	0.352	CT vs. CC/TT	0.308	0.154	0.615	0.0006
RD – M	rs1050828	G6PD	C/T	0.193	0.203	T vs. C	1.035	0.496	2.160	0.9280
RD – F	rs1050828	G6PD	C/T	0.211	0.164	CT vs. CC/TT	0.460	0.214	0.989	0.0398
Acid – M	rs1050829	G6PD	T/C	0.371	0.362	C vs. T	1.024	0.632	1.659	0.9227
Acid – F	rs1050829	G6PD	T/C	0.400	0.335	CT vs. CC/TT	0.345	0.205	0.580	3.71E-05
Acid – M	rs1050828	G6PD	C/T	0.193	0.138	T vs. C	0.781	0.419	1.453	0.4319
Acid – F	rs1050828	G6PD	C/T	0.211	0.158	CT vs. CC/TT	0.495	0.279	0.879	0.0145

SM = severe malaria, SMA = severe malarial anaemia, CM = cerebral malaria, RD = respiratory distress, Acid = acidosis, MinA = minor allele, MajA = major allele, ConMAF = minor allele frequency in controls, CaseMAF = minor allele frequency in cases, OR = odds ratio, 95% Confidence interval (LCL, UCL), P = P-value; for X chromosome SNPs (rs1126535 (CD40), rs1050829 (G6PD-376), rs1050828 (G6PD-202/A-), analyses are presented for separately for females (F) and males (M), NA not applicable, *.

**Table 3:** Interaction between alpha-thalassaema and HbS and severe malaria.

Phenotype	HbS/thal	OR**	LCL	UCL	P
SM	AA/AA	1.000			
SM	AA/AB	0.945	0.687	1.301	0.7292
SM	AA/BB	0.587	0.342	1.004	0.0517
SM	AS/AA	0.115	0.039	0.340	<0.0001
SM	AS/AB	0.092	0.031	0.275	<0.0001
SM	AS/BB	0.115	0.014	0.976	0.0475
SMA	AA/AA	1.000			
SMA	AA/AB	0.779	0.524	1.157	0.2162
SMA	AA/BB	0.413	0.203	0.839	0.0145
SMA	AS/AA	0.150	0.042	0.528	0.0031
SMA	AS/AB	0.126	0.036	0.437	0.0011
SMA	AS/BB	-	-	-	-

* No significant evidence of a statistical interaction P>0.4, OR = odds ratio, 95% Confidence interval (LCL, UCL), P = P-value, SM = severe malaria, SMA = severe malarial anaemia,

** adjusted for age, gender and ethnicity.

Children carrying either the RTN3 (rs 542998 TC vs. CC OR 1.781, 95% CI 1.276–2.487, p = 0.0006) or the IL-13 (rs 20541 CT vs. TT OR 2.02, 95% CI 1.346–3.03, p = 0.0008) SNPs were more likely than other children to develop CM (Table 2, Figure 2). Furthermore, heterozygosity for the rs2230739 SNP in the ADCY9 gene, encoding an adenylate cyclase, was associated with a 50% reduction in the risk of acidosis (AG vs. AA/GG, OR = 0.516, 95% CI 0.302–0.882, p = 0.0129) (Table 2, Figure 2).

Two mutations in the G6PD gene on the X chromosome (202: rs1050828 CT vs. other and 376: rs1050829 CT vs. other) were strongly associated with protection from severe malaria, and with protection from each of the different clinical phenotypes of severe malaria, in heterozygous females (OR = 0.3–0.5; p<0.05–p<8e-6) but not in hemizygous males (Table 2). The protection was best explained in the dominant model inheritance of the trait (supplementary Table 2). However, a haplotype analysis using both G6PD markers (202/376: CT [B - enzymatic group], CC [A+ deficiency], and TC [A- deficiency]), indicated no strong evidence of association with protection (P>0.1; supplementary table 4). Interestingly, polymorphisms in another X-linked gene, namely that encoding the T cell co-stimulatory receptor CD40 ligand (CD40L; CD154), were associated with either an increased (rs3092945: OR (TT vs. CT/CC) = 5.27, 95% CI 1.491–18.621, p = 0.01) or a decreased (rs1126535; OR (TT vs. CT/CC) = 0.331, 95% CI 0.143–0.766, p = 0.006) risk of respiratory distress (supplementary table 3). The haplotypic analysis indicates that having CT haplotypes for rs3092945 and rs1126535 was a risk of developing respiratory distress in females (OR = 1.768, 95%CI 1.027–3.043, p = 0.040; supplementary table 5).

Polymorphisms in three other genes of known immunological function were also associated with altered risk of severe malaria. Carriage of the rs17411697 SNP in the gene encoding IL-1α was associated with an increased risk of acidosis (GT or TT vs. GG, OR = 1.681, 95% CI 1.185–2.382, p = 0.0035), whereas carriage of the rs1800890 SNP in the IL-10 gene was associated with a decreased risk of severe malaria (additive T, OR = 0.747, 95% CI 0.588–0.949, p = 0.0165) but not with any of the individual severe malaria syndromes. Carriage of the rs7935564 SNP in the gene encoding TRIM5 (an E3 ubiquitin-ligase) was associated with an increased risk of severe malaria (additive A, OR = 1.273, 95% CI 1.042–1.555, p = 0.02).

## Discussion

We set out to investigate whether genetic polymorphisms previously linked to altered risk of severe malaria, or implicated in resistance to other acute infections, were linked to risk of severe malaria in children living in the Tanga region of Tanzania. For some of these polymorphisms, there have been discrepancies in published findings, and these could be a result of variation in phenotype definition, sample size differences, choice of controls, village surveys versus hospital-based studies, immune status, differences in linkage disequilibrium patterns between populations [29], and potentially functional polymorphisms being distal from candidate genes and polymorphisms genotyped [30]. To maximise the robustness of any associations we detected, we standardised recruitment procedures using case report forms, pre-defined the criteria for diagnosis of severe malaria and various severe malaria syndromes. In addition, all analyses were adjusted for the ethnicity of the child and their ward of residence, thereby minimising confounding effects and potential false positives arising from population stratification.

The study confirmed the known (∼90%) reduction in severe malaria risk conferred by the haemoglobin B (sickle cell) AS genotype [10]. The low frequency of the S allele in the control children (∼4.9%) is in keeping with other populations (see http://www.map.ox.ac.uk/) in West (Burkina Faso 5.2%, Cameroon 6.5%, Gambia 7.6%, Ghana 6.5%, Mali 3.8%) and East Africa (Kenya 6.4%, Malawi 2.7%).

The α^+^-thalassaemia polymorphism was also associated with severe malaria anaemia, confirming observations in other studies [9], [31]. This can be explained by the observation that in α-thalassaemics the mechanical destruction of infected red blood cells that leads to anaemia is slowed or prevented [32]. Similarly, a recent study in Papua New Guinea [31] found that homozygous α^+^- thalassaemia children are protected from the risk of severe malarial anaemia by a lower reduction in haemoglobin concentration compared to children of wild-type genotype through increased erythrocyte count and microcytosis.

The study also confirmed the well-described association between X-linked G6PD deficiency and protection from severe malaria. The geographical distribution of G6PD deficiency offers strong evidence for its selection by malaria; increased oxidative stress in G6PD-deficient red blood cells is assumed to reduce parasite replication and thereby confer protection (reviewed in [8]). Some studies have reported that both hemizygous males and heterozygous females are protected [33] whereas other studies suggest either that only male hemizygotes are protected [11], [34] or that, as observed here, protection is limited to female heterozygotes [35]. These discrepancies may be due to allelic/haplotypic heterogeneity and more detailed resolution of individual alleles will be required to identify true causal relationships [36].

Interestingly, we found evidence that polymorphisms in another X-linked gene (CD40L) were associated with altered risk of respiratory distress, and that a particular CD40L haplotype was particularly associated with increased disease risk in females. By contrast, in The Gambia, males hemizygous for the CD40L–726C (rs17424229) had a reduced risk of developing severe malaria [17]. CD40-L is a stimulatory co-receptor expressed by activated CD4+ T cells, and plays a pivotal role in augmenting T cell mediated immune function. T cell hypo-responsiveness may predispose to failure to clear an infection whereas hyper-responsiveness may lead to immunopathology.

Polymorphism in the adenyl cyclase 9 gene (ADCY9-rs2230739A>G in a heterozygous model) was associated with decreased risk of acidosis. Adenyl cyclase 9 is a component of the stimulatory G protein (Gs) pathway. Inhibition of the Gs signaling pathway in human erythrocytes blocks merozoite invasion and inhibits intracellular parasite maturation [37]suggesting that mutations in genes within this pathway might plausibly be linked to malaria outcomes [38]. Although there was no evidence that adenyl cyclase 9 polymorphisms were associated with severe malaria outcomes in a large study of cases from Malawi and The Gambia [39], SNPs in two other genes in the Gs signal transduction pathway - adenosine receptor 2 (ADORA2A) and G-alpha-s (GNAS) - have been linked to altered risk of severe malaria in multicentre studies in The Gambia, Malawi and Kenya [39], [40]. Data from studies on chronic acidosis suggest that the high proton concentrations exerted by acidosis stimulate proton sensitive G-protein-coupled receptors, which are mediated by the cellular cAMP/PKA pathway and it is possible that mutations in Gs would modify this effect [41]. Taken together, these studies suggest that modulation of the Gs signal transduction pathway may modulate the outcome of malaria infection. The number of different associations detected, across different SNPs and loci, suggest that this pathway may be a biologically relevant modulator of host susceptibility to malaria.

The role of IL-10 in limiting the pathological consequences of malaria-induced inflammation is well established (reviewed in [42]). IL-10 deficient mice infected with normally resolving malaria infections succumb to acute immunopathology [43], high ratios of serum IL-10 to proinflammatory cytokines such as IFN-γ, TNF and IL-12 are associated with positive clinical outcomes [44], [45], [46] and IL-10-secreting T cells have been correlated with resistance to severe malaria pathology [47], [48]. Furthermore, polymorphisms involving the IL-10 promoter region have been associated with both severe malaria outcomes and IL-10 production levels in Kenya [49] and Mozambique [50], and polymorphisms in the IL-10 receptor gene promoter have also been linked to protection against severe malaria in Gabon [51], but the results of a family based study in The Gambia raised questions as to whether associations with IL-10 signalling pathways might be confounded by foetal survival rates or other sources of inheritance bias [26]. Nevertheless, the evidence from the present study reinforces IL-10 signalling as a pathway of interest for regulating the severe outcomes of malaria infection.

In this study, carriage of the rs20541C>T polymorphism was associated with increased risk of developing cerebral malaria; this polymorphism introduces a nonsynonymous change in exon 4 of the gene encoding IL-13. In line with the known role of IL-13 in induction of Th2 responses, rs20541polymorphisms have also been linked to allergy and autoimmune disease [52], [53] as well as altered outcomes of helminth (Schistosome spp) infections [54]. Our data support other studies implicating IL-13 polymorphisms with risk of severe malaria in Thai adults [55], [56] and associations between 5q31–q33 haplotypes (which span the IL13 locus) and antimalarial antibody responses [57]; the recurrent link between IL-13 and risk of severe malaria would seem to warrant further investigation.

TRIM5 (an E3 ubiquitin ligase) is an innate immune signalling molecule that activates pro-inflammatory signalling pathways leading to activation of NF-κB and AP-1 [58]. Mutations in TRIM5 have been associated with HIV susceptibility in different populations (reviewed in [59]), presumably explained by the ability of TRIM5α to bind HIV virions and target them for proteosomal destruction [60], but have not previously been linked to malaria susceptibility.

We observed associations between polymorphisms in reticulon 3 (RTN3- rs542998) and increased risk of cerebral malaria. Reticulon 3 is a ubiquitously expressed endoplasmic reticulum protein and is believed to be involved in membrane trafficking in the early secretory pathway [61]. It is of interest that RTN3 expression has been repeatedly associated with neurodegenerative diseases in humans [62] and that RTN3 is differentially expressed in brains of experimental malaria resistant and susceptible strains of mice infected with *Plasmodium berghei* ANKA [63].

## Conclusions

This study confirms previously known genetic associations with protection from severe malaria (HbS, G6PD and α^+^- thalassaemia). The study also identifies two X-linked genes associated with altered risk of severe malaria in females, identifies mutations in ADCY9, IL1A and CD40L as being associated with altered risk of severe respiratory distress and acidosis, both of which are characterised by high serum lactate levels, and also identifies two novel (IL-13 and RTN3) genetic associations with cerebral malaria. The RTN3 and TRIM5 associations draw attention to the potential role of intracellular protein trafficking and degradation in the pathogenesis of severe malaria but further studies are needed in areas of differing malaria epidemiology to replicate these findings and understand their functional consequences. The testing of multiple data sets, from diverse malaria endemic regions, within the MalariaGEN consortium offers a powerful route to validate these genetic associations [64].

## Supporting Information

Table S1 List of all candidate single nucleotide polymorphisms.
(DOCX)

Table S2 G6PD associations with malaria phenotypes.
(DOCX)

Table S3 CD40LG associations with malaria phenotypes.
(DOCX)

Table S4 Haplotypic analysis of G6PD.
(DOCX)

Table S5 Haplotypic analysis of CD40LG.
(DOCX)
