# Supplementary material for: Candidate Human Genetic Polymorphisms and Severe Malaria in a Tanzanian Population
Source: PLoS One. 2012 Oct 29;7(10):e47463. doi: 10.1371/journal.pone.0047463 (PMC3483265; doi:10.1371/journal.pone.0047463)
Supplement: Table S2 — G6PD associations with malaria phenotypes. (DOCX) [file pone.0047463.s002.docx]

**Table S2**

**G6PD associations with malaria phenotypes**

| Phenotype | SNP | Gene | Maj/  Min | Con  MAF | Case  MAF | Comparison | OR | LCL | UCL | P |
| --- | --- | --- | --- | --- | --- | --- | --- | --- | --- | --- |
| SM - M | rs1050829 | G6PD | T/C | 0.371 | 0.366 | C vs. T | 1.003 | 0.667 | 1.509 | 0.9885 |
| SM – F | rs1050829 | G6PD | T/C | 0.400 | 0.340 | CC/CT vs. TT | 0.495 | 0.327 | 0.750 | 0.0008 |
| SM - M | rs1050828 | G6PD | C/T | 0.193 | 0.146 | T vs. C | 0.770 | 0.458 | 1.293 | 0.3228 |
| SM - F | rs1050828 | G6PD | C/T | 0.211 | 0.174 | TT/CT vs. CC | 0.629 | 0.406 | 0.973 | 0.0363 |
| SMA - M | rs1050829 | G6PD | T/C | 0.371 | 0.389 | C vs. T | 1.055 | 0.643 | 1.729 | 0.8325 |
| SMA - F | rs1050829 | G6PD | T/C | 0.400 | 0.295 | CC/CT vs. TT | 0.303 | 0.171 | 0.536 | 2.74E-05 |
| SMA - M | rs1050828 | G6PD | C/T | 0.193 | 0.170 | T vs. C | 0.926 | 0.500 | 1.713 | 0.8052 |
| SMA - F | rs1050828 | G6PD | C/T | 0.211 | 0.139 | TT/CT vs. CC | 0.383 | 0.202 | 0.725 | 0.0022 |
| CM - M | rs1050829 | G6PD | T/C | 0.371 | 0.404 | C vs. T | 1.185 | 0.594 | 2.365 | 0.6314 |
| CM - F | rs1050829 | G6PD | T/C | 0.400 | 0.391 | CC/CT vs. TT | 0.626 | 0.308 | 1.273 | 0.1987 |
| CM - M | rs1050828 | G6PD | C/T | 0.193 | 0.149 | T vs. C | 0.681 | 0.271 | 1.709 | 0.4009 |
| CM - F | rs1050828 | G6PD | C/T | 0.211 | 0.211 | TT/CT vs. CC | 0.818 | 0.391 | 1.711 | 0.5908 |
| RD - M | rs1050829 | G6PD | T/C | 0.371 | 0.458 | C vs. T | 1.369 | 0.748 | 2.505 | 0.3101 |
| RD - F | rs1050829 | G6PD | T/C | 0.400 | 0.352 | CC/CT vs. TT | 0.411 | 0.210 | 0.804 | 0.0090 |
| RD - M | rs1050828 | G6PD | C/T | 0.193 | 0.203 | T vs. C | 1.035 | 0.496 | 2.160 | 0.9280 |
| RD - F | rs1050828 | G6PD | C/T | 0.211 | 0.164 | TT/CT vs. CC | 0.584 | 0.289 | 1.181 | 0.1285 |
| Acid - M | rs1050829 | G6PD | T/C | 0.371 | 0.362 | C vs. T | 1.024 | 0.632 | 1.659 | 0.9227 |
| Acid - F | rs1050829 | G6PD | T/C | 0.400 | 0.335 | CC/CT vs. TT | 0.433 | 0.259 | 0.723 | 0.0012 |
| Acid - M | rs1050828 | G6PD | C/T | 0.193 | 0.138 | T vs. C | 0.781 | 0.419 | 1.453 | 0.4319 |
| Acid – F | rs1050828 | G6PD | C/T | 0.211 | 0.158 | TT/CT vs. CC | 0.553 | 0.321 | 0.952 | 0.0304 |

SM = severe malaria, CM = cerebral malaria, SMA = severe malarial anaemia, RD = respiratory distress, Acid = acidosis, MinA = minor allele, MajA = major allele, ConMAF = minor allele frequency in controls, CaseMAF = minor allele frequency in cases, OR = odds ratio, 95% Confidence interval (LCL, UCL), P = P-value; for X chromosome SNPs (rs1050829 (G6PD-376), rs1050828 (G6PD-202/A-), analyses are presented for separately for females (F) and males (M), NA not applicable, *
