# Supplementary material for: Candidate Human Genetic Polymorphisms and Severe Malaria in a Tanzanian Population
Source: PLoS One. 2012 Oct 29;7(10):e47463. doi: 10.1371/journal.pone.0047463 (PMC3483265; doi:10.1371/journal.pone.0047463)
Supplement: Table S3 — CD40LG associations with malaria phenotypes. (DOCX) [file pone.0047463.s003.docx]

**Table S3**

**CD40LG associations with malaria phenotypes**

| Phenotype | SNP | Gene | Maj/  Min | Con  MAF | Case  MAF | Comparison | OR | LCL | UCL | P |
| --- | --- | --- | --- | --- | --- | --- | --- | --- | --- | --- |
| SM - M | rs3092945 | CD40LG | T/C | 0.223 | 0.215 | C vs. T | 0.917 | 0.576 | 1.461 | 0.7167 |
| SM – F | rs3092945 | CD40LG | T/C | 0.205 | 0.271 | CC vs. CT/TT | 2.315 | 0.970 | 5.523 | 0.052 |
| SM - M | rs1126535 | CD40LG | T/C | 0.209 | 0.237 | C vs. T | 1.282 | 0.803 | 2.047 | 0.2965 |
| SM - F | rs1126535 | CD40LG | T/C | 0.238 | 0.184 | CC/CT vs. TT | 0.649 | 0.425 | 0.990 | 0.044 |
| SMA - M | rs3092945 | CD40LG | T/C | 0.223 | 0.213 | C vs. T | 0.880 | 0.504 | 1.536 | 0.6510 |
| SMA - F | rs3092945 | CD40LG | T/C | 0.205 | 0.247 | CC vs. CT/TT | 2.529 | 0.797 | 8.019 | 0.120 |
| SMA - M | rs1126535 | CD40LG | T/C | 0.209 | 0.264 | C vs. T | 1.516 | 0.874 | 2.629 | 0.1389 |
| SMA - F | rs1126535 | CD40LG | T/C | 0.238 | 0.177 | CC/CT vs. TT | 0.540 | 0.300 | 0.972 | 0.0367 |
| CM - M | rs3092945 | CD40LG | T/C | 0.223 | 0.231 | C vs. T | 1.085 | 0.510 | 2.307 | 0.8323 |
| CM - F | rs3092945 | CD40LG | T/C | 0.205 | 0.267 | CC vs. CT/TT | 1.156 | 0.215 | 6.229 | 0.8668 |
| CM - M | rs1126535 | CD40LG | T/C | 0.209 | 0.216 | C vs. T | 1.158 | 0.528 | 2.542 | 0.7164 |
| CM - F | rs1126535 | CD40LG | T/C | 0.238 | 0.178 | CC/CT vs. TT | 0.723 | 0.340 | 1.535 | 0.3938 |
| RD - M | rs3092945 | CD40LG | T/C | 0.223 | 0.189 | C vs. T | 0.793 | 0.377 | 1.667 | 0.5372 |
| RD - F | rs3092945 | CD40LG | T/C | 0.205 | 0.318 | CC vs. CT/TT | 5.270 | 1.491 | 18.621 | 0.0096 |
| RD - M | rs1126535 | CD40LG | T/C | 0.209 | 0.237 | C vs. T | 1.301 | 0.634 | 2.670 | 0.4755 |
| RD - F | rs1126535 | CD40LG | T/C | 0.238 | 0.159 | CT vs. CC/TT | 0.361 | 0.169 | 0.772 | 0.0062 |
| Acid - M | rs3092945 | CD40LG | T/C | 0.223 | 0.235 | C vs. T | 1.070 | 0.627 | 1.830 | 0.8034 |
| Acid - F | rs3092945 | CD40LG | T/C | 0.205 | 0.246 | CC vs. CT/TT | 2.147 | 0.749 | 6.157 | 0.1539 |
| Acid - M | rs1126535 | CD40LG | T/C | 0.209 | 0.242 | C vs. T | 1.284 | 0.744 | 2.216 | 0.3704 |
| Acid – F | rs1126535 | CD40LG | T/C | 0.238 | 0.201 | CC/CT vs. TT | 0.775 | 0.460 | 1.306 | 0.3366 |

SM = severe malaria, CM = cerebral malaria, SMA = severe malarial anaemia, RD = respiratory distress, Acid = acidosis, MinA = minor allele, MajA = major allele, ConMAF = minor allele frequency in controls, CaseMAF = minor allele frequency in cases, OR = odds ratio, 95% Confidence interval (LCL, UCL), P = P-value; analyses are presented for separately for females (F) and males (M), NA not applicable, *
