# Supplementary material for: Candidate Human Genetic Polymorphisms and Severe Malaria in a Tanzanian Population
Source: PLoS One. 2012 Oct 29;7(10):e47463. doi: 10.1371/journal.pone.0047463 (PMC3483265; doi:10.1371/journal.pone.0047463)
Supplement: Table S4 — Haplotypic analysis of G6PD. (DOCX) [file pone.0047463.s004.docx]

**Table S4**

**Haplotypic analysis of G6PD***

|  | Phenotype | Haplotype | Frequency | OR | 95% CI | | P |
| --- | --- | --- | --- | --- | --- | --- | --- |
|  |  | *202/376* |  |  |  |  |  |
| Male | *SM* | CT [B] | 0.627 | 1.000 |  |  |  |
|  |  | CC [A] | 0.201 | 1.110 | 0.926 | 1.430 | 0.418 |
|  |  | TC [A-] | 0.172 | 0.908 | 0.712 | 1.205 | 0.568 |
| Female | *SM* | CT [B] | 0.628 | 1.000 |  |  |  |
|  |  | CC [A] | 0.177 | 0.756 | 0.558 | 1.158 | 0.151 |
|  |  | TC [A-] | 0.195 | 0.754 | 0.529 | 1.063 | 0.128 |
| Overall | *SM* | CT [B] | 0.628 | 1.000 |  |  |  |
|  |  | CC [A] | 0.189 | 1.000 | 0.813 | 1.229 | 0.997 |
|  |  | TC [A-] | 0.183 | 0.859 | 0.695 | 1.062 | 0.160 |
|  |  |  |  |  |  |  |  |
| Male | *SMA* | CT [B] | 0.618 | 1.000 |  |  |  |
|  |  | CC [A] | 0.194 | 1.085 | 0.796 | 1.478 | 0.607 |
|  |  | TC [A-] | 0.188 | 1.009 | 0.739 | 1.378 | 0.954 |
| Female | *SMA* | CT [B] | 0.629 | 1.000 |  |  |  |
|  |  | CC [A] | 0.179 | 0.614 | 0.357 | 1.058 | 0.079 |
|  |  | TC [A-] | 0.192 | 0.456 | 0.262 | 0.793 | 0.006 |
| Overall | *SMA* | CT [B] | 0.624 | 1.000 |  |  |  |
|  |  | CC [A] | 0.187 | 0.966 | 0.742 | 1.259 | 0.801 |
|  |  | TC [A-] | 0.190 | 0.815 | 0.621 | 1.069 | 0.140 |

* adjusted for age and ethnicity, SM = severe malaria, SMA = severe malaria anaemia
