# Supplementary material for: Candidate Human Genetic Polymorphisms and Severe Malaria in a Tanzanian Population
Source: PLoS One. 2012 Oct 29;7(10):e47463. doi: 10.1371/journal.pone.0047463 (PMC3483265; doi:10.1371/journal.pone.0047463)
Supplement: Table S5 — Haplotypic analysis of CD40LG. (DOCX) [file pone.0047463.s005.docx]

**Table S5**

**Haplotypic analysis of CD40LG**

|  | Phenotype | Haplotype | Frequency | OR | 95% CI | | P |
| --- | --- | --- | --- | --- | --- | --- | --- |
|  |  | *rs3092945/*  rs1126535 |  |  |  |  |  |
| Male | *SM* | TT | 0.552 | 1.000 |  |  |  |
|  |  | CT | 0.220 | 0.984 | 0.773 | 1.254 | 0.899 |
|  |  | TC | 0.228 | 1.120 | 0.881 | 1.424 | 0.356 |
| Female | *SM* | CT | 0.551 | 1.000 |  |  |  |
|  |  | CC | 0.237 | 1.209 | 0.856 | 1.707 | 0.282 |
|  |  | TT | 0.212 | 0.770 | 0.541 | 1.096 | 0.148 |
| Overall | *SM* | TT | 0.551 | 1.000 |  |  |  |
|  |  | CT | 0.228 | 1.099 | 0.906 | 1.332 | 0.338 |
|  |  | TC | 0.220 | 0.983 | 0.811 | 1.192 | 0.862 |
|  |  |  |  |  |  |  |  |
| Male | *RD* | TT | 0.562 | 1.000 |  |  |  |
|  |  | CT | 0.216 | 0.901 | 0.610 | 1.331 | 0.600 |
|  |  | TC | 0.222 | 1.116 | 0.771 | 1.617 | 0.564 |
| Female | *RD* | TT | 0.549 | 1.000 |  |  |  |
|  |  | CT | 0.228 | 1.768 | 1.027 | 3.043 | 0.040 |
|  |  | TC | 0.223 | 0.620 | 0.341 | 1.127 | 0.118 |
| Overall | *RD* | TT | 0.555 | 1.000 |  |  |  |
|  |  | CT | 0.222 | 1.091 | 0.810 | 1.470 | 0.566 |
|  |  | TC | 0.223 | 0.877 | 0.645 | 1.191 | 0.400 |

* adjusted for age and ethnicity, SM = severe malaria, RD = respiratory distress
